# Atomistic Simulation of Collapse and Recrystallization in Hollow Gold and Silver Nanoparticles with Ultrathin, Porous Walls

**DOI:** 10.1021/acsphyschemau.6c00001

**Published:** 2026-04-05

**Authors:** Vladimir S. Myasnichenko, Denis Sokolov, Anatolii Bazulev, Kseniya Savina, Oleg Polev, Valentin Romanovski, Nickolay Sdobnyakov

**Affiliations:** † Department of General Physics, 64970 Tver State University, Tver 170100, Russia; ‡ Department of Materials Science and Engineering, 2358 University of Virginia, Charlottesville, Virginia 22904, United States

**Keywords:** gold and silver nanoframes, Monte-Carlo simulation, tight-binding potential, structure formation, crystallization, recrystallization, collapse

## Abstract

Understanding the thermal stability of hollow metal nanoframes remains a significant challenge due to strongly coupled effects such as porosity, curvature, and defect migration. Using tight-binding Monte Carlo simulations, we systematically investigate gold and silver nanoframes with cavity radii of 7.0–19.9 Å and quantitatively resolve their structural evolution through a shape-parametrization method that tracks cavity collapse and global flattening with ångström-level precision. Silver nanoframes exhibit lateral pore closure at 605–785 K, cavity collapse at 783–844 K, and melting near 852–900 K. Gold analogues show earlier pore closure (585–720 K) but higher collapse temperatures (688–823 K) and melting at 772–825 K, consistent with more coordinated recrystallization. The flattening parameter rises to 0.18–0.22 before global deformation, serving as a universal geometric indicator. Together, these quantitative criteria advance mechanistic understanding and enable the predictive design of thermally robust porous metal nanostructures.

## Graphical abstract

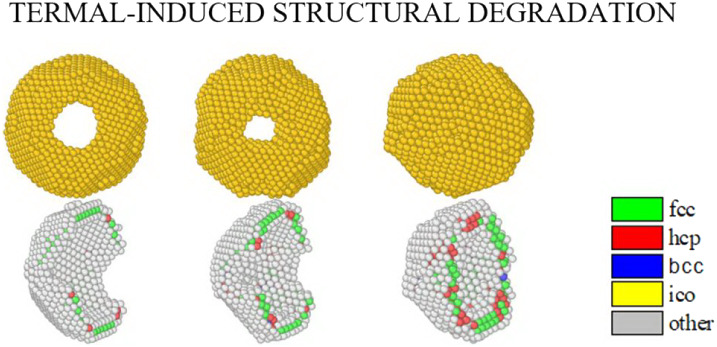

## 1 Introduction

Hollow noble-metal nanostructures with hierarchical porosity—such as nanoframes and nanocages—have emerged as versatile platforms for catalysis, plasmonics, sensing, and photothermal energy conversion because they combine extremely high surface-to-volume ratios with tunable electromagnetic and chemical functionality. Nanoframes consist of interconnected, ultrathin ridges without continuous facets, whereas nanocages exhibit closed or semiclosed shells that may retain internal porosity.^1,2^ More complex architectures, including nested “matryoshka”-type assemblies^3^ and dual-rim three-dimensional frameworks,^4^ further expand the accessible design space. A defining feature across these systems is the presence of ultrathin walls and localized regions of high curvature that amplify surface energy and defect concentration. Such structural characteristics are directly linked to catalytic activity,^5–7^ plasmonic field enhancement,^8,9^ and SERS performance,^10^ yet they also introduce intrinsic thermodynamic instability.

Metallic nanoframes are commonly synthesized by template-based strategies, including sacrificial-template dealloying and galvanic replacement of Ag nanocrystals with more noble metals such as Au, Pt, or Pd.^11–13^ The resulting structures feature adjustable wall thickness and porosity controlled by temperature, alloy composition, and etchant chemistry.^6,14^ Additional morphologies may be generated through self-assembly routes, including amorphous precursor aggregation.^15^ Despite this synthetic versatility, the thermodynamic stability of thin-walled frames remains a persistent limitation: even modest heating can trigger pore coalescence, wall buckling, structural collapse, and a loss of functional properties.^16,17^

Prior experimental and computational studies have shown that thermal stability depends sensitively on the wall thickness, porosity, and curvature of local features. For instance, increasing the wall thickness of Ag–Au nanocages from 3.8 to 13 nm substantially raises their collapse temperature,^16^ and similar trends have been observed in spherical hollow particles,^2^ cubic nanoframes,^6^ and Pd@Pt architectures exhibiting defect-mediated corrosion pathways.^18^ Porosity, in particular, has been shown to lower the transition temperature to partially collapsed states, yet quantitative guidelines for the interplay between porosity, curvature, and collapse dynamics remain limited. Existing observations suggest that regions of high curvature or low coordination can act as defect sources or vacancy traps, altering recrystallization pathways, but these mechanisms have not been comprehensively resolved at the atomistic level.^19–22^

Several critical knowledge gaps therefore remain. First, the role of pore geometry and surface curvature in controlling defect redistribution and recrystallization is not fully understood. Second, no universal stability criteria exist for hollow or frame-like architectures, particularly those with ultrathin walls and hierarchical porosity. Third, although composition-dependent effects have been reported in Au/Ag alloy nanostructures,^2^ the atomistic origin of material-specific relaxation behavior remains insufficiently explored. These gaps hinder the predictive design of nanoframes for applications in catalysis, electrocatalysis, surface-enhanced spectroscopy, and photothermal energy conversion.

Atomistic simulations offer a route to isolate the underlying thermodynamic driving forces governing stability. Previous computational work has used caloric curves (temperature dependences), local-density analysis, and structural fingerprinting to study the collapse of hollow nanoshells,^2^ but the geometric complexity of porous frames presents additional challenges. In particular, conventional surface triangulation methods struggle to quantify subtle deformation modes, identify pore-closure events, and classify shape transitions during thermal loading. Moreover, most existing simulations lack a unified geometric descriptor capable of capturing both local and global deformations in a manner transferable across materials and frame dimensions.

In this work, we address these challenges through large-scale atomistic Monte Carlo simulations combined with a robust geometric reconstruction framework. Initial nanoframe configurations were generated and optimized using the ClusterEvolution software,^23^ followed by thermal evolution simulations in the Metropolis environment^24,25^ using a tight-binding potential for Au and Ag,^26^ which ensures the physical realism of the model. The Metropolis method used in this study was aimed at finding thermodynamically stable configurations and qualitatively describing structural transitions; this was the primary goal of the work, not an accurate reproduction of the experimental time scale. The Monte Carlo method (Metropolis algorithm) has proven itself to be a good candidate for studying structural transformations and the thermodynamic characteristics of nanoparticles.^27–30^ The maximum step (per component) by which an atom can shift is selected: stepmax ∼0.3 Å. A uniform random variable is generated within the range [0, stepmax], resulting in a vector [d*x*, d*y*, d*z*] and shifted relative to the initial position by this vector. An NVT ensemble is used. The simulation box size is 23 Å. We performed 100,000,000 MC steps, which allowed the system to transcend local oscillations and demonstrate real morphological changes. This allows us to observe rearrangements involving defects. It must be acknowledged that, unlike the molecular dynamics method, the Monte Carlo method (Metropolis scheme) does not contain a time scale. It should be noted that the equivalence of the Monte Carlo method and the molecular dynamics method is based on the ergodic hypothesis (introduced by Boltzmann), which states that the time average (the molecular dynamics method) is equal to the ensemble average (the Monte Carlo method). Its justifications were made quite a long time ago.^31^ At the same time, both methods are currently actively used to describe structural transformations,^32–34^ confirming the adequacy and possibility of using either of them with the correct setting of experimental conditions and selection of the interaction potential. Previously, the Monte Carlo method was successfully used by us to study the processes of structural optimization of both monometallic gold and silver nanoparticles, and for binary nanosystems.^29,35–38^

Structural transformations were characterized using polyhedral template matching (PTM),^39^ and common-neighbor analysis (CNA),^40^ which have complementary strengths for identifying crystalline motifs in complex nanostructures.^41^ OVITO-based visualization^42^ enabled tracking of local ordering, pore healing, and defect evolution throughout the thermal cycle.

A central methodological contribution of this study is a shape-parametrization algorithm that reconstructs a nanoframe’s meridional profile from three-dimensional atomic coordinates by projecting atomic positions onto a two-dimensional plane, performing binning along the symmetry axis, and applying spline-based contour reconstruction. This approach, which draws on techniques used in tomography,^43,44^ mesh processing,^45^ and medical imaging,^46^ provides smooth, noise-robust profiles from which cavity radius, pore dimensions, wall thickness, surface curvature, cross-sectional area, and perimeter can be calculated. By mapping these parameters over temperature, we classify nanoframe states (classic, concave, closed, and collapsed) and identify collapse thresholds. Importantly, the framework is general and does not rely on specific material properties, enabling application to a wide range of hollow or porous nanostructures.

The integration of atomistic simulations with a universal geometric descriptor allows us to quantitatively resolve the hierarchical relaxation processes that determine stability. We identify temperature-dependent pore-closure events, cavity-collapse thresholds, changes in crystallinity, and material-specific relaxation modes across nanoframe sizes spanning internal radii from 7.0 to 19.9 Å (or a constant 2700 atoms for every size). Silver nanoframes exhibit rapid, weakly coordinated collapse driven by curvature-amplified defect migration, whereas gold nanoframes undergo more gradual, cooperative recrystallization that stabilizes the structure over a wider temperature range. The flattening parameter emerges as a universal indicator of global deformation, reflecting the buildup of anisotropic stress and predicting imminent collapse across all geometries examined.

By linking microscopic atomic rearrangements to macroscopic shape evolution, this work establishes predictive stability criteria for hollow noble-metal nanoframes and provides quantitative design principles for tailoring thermal robustness through geometry, material choice, and porosity. These insights directly support the rational development of nanoframes for catalytic, sensing, plasmonic, and photothermal applications. In contrast to existing approaches based on surface triangulation or local-density segmentation,^47,48^ our method introduces a material-independent geometric parametrization scheme that allows direct tracking of pore closure and cavity collapse with ångström-level precision throughout thermal loading. The key methodological novelty lies in (i) explicit reconstruction of meridional profiles from atomistic coordinates, (ii) metric quantification of cavity/pore evolution, and (iii) applicability to a broad class of hollow and porous nanostructures without assumptions about crystallinity or material type. This closes an important methodological gap in quantifying collapse dynamics in ultrathin nanoframes.

## 2 Methodology of Atomistic Simulation and Structural Analysis

### 2.1 Atomistic Simulation and Thermodynamic Analysis

Atomistic simulations were conducted to resolve the thermally driven relaxation and collapse mechanisms in hollow noble-metal nanoframes. The computational workflow expands upon previously established approaches for hollow nanoshell stability analysis,^2^ where structural transitions were primarily identified through changes in local atomic density. Initial nanoframe geometries with prescribed internal radii were generated and preoptimized using the ClusterEvolution (CE) package,^23^ which provides energy minimization under tight-binding interactions. It should also be noted that the tight-binding potential is not the only possible one; the potential used within the embedded atom method is widely employed. However, the choice of the interaction potential remains open for discussion.

Thermal evolution was modeled using the Metropolis simulation environment,^24^ which implements classical Monte Carlo sampling based on the Metropolis acceptance criterion.^25^ Interatomic forces were described using the widely validated tight-binding potential for transition metals.^26^ Parameter values used for Au–Au and Ag–Ag interactions are given in Table 1. For each configuration, the system was heated from 300 to 1200 K in increments of 10 K. At each temperature, the system was equilibrated over 9 × 10^7^ Monte Carlo steps, followed by 10^8^ steps for statistical averaging of thermodynamic observables. This extensive sampling ensures that the system explores the available energy states and overcomes local barriers.

**Table 1:** Parameters of the Tight-Binding Potential

Bond type	*A*, eV	ζ, eV	*p*	*q*	*r*_0_, Å	*r*_cutoff_, Å
*A*_u_	0.2061	1.790	10.229	4.036	2.8838	7.55
*A*_g_	0.1028	1.178	10.928	3.139	2.8890	7.55

Throughout the simulation, several global descriptors were monitored, including the internal cavity radius, external dimensions, radius of gyration, and specific potential energy. Caloric curves obtained from temperature-dependent potential energy were used to identify characteristic transitions, such as pore closure, cavity collapse, and melting. This methodology follows established practice for analyzing hollow nanostructure stability,^2,49^ enabling the identification of thermodynamic signatures corresponding to structural rearrangements.

Structural evolution and local crystallinity were analyzed using OVITO^42^ equipped with polyhedral template matching (PTM)^39^ and common-neighbor analysis (CNA)^40^ routines. These two complementary approaches enable robust detection of fcc, hcp, bcc, and icosahedral motifs in nanoparticles with complex porosity.^41^ OVITO was also used to verify pore healing and visualize changes in the internal surfaces across nanoframes of different sizes.

While topological descriptors capture local ordering, a detailed analysis of collapse dynamics requires quantitative shape tracking. As collapse is governed by geometry—specifically, curvature evolution and pore constriction—a dedicated method for shape parametrization was implemented. Integrating topological and geometric analysis enables precise mapping of the sequence of pore closure, cavity shrinkage, and global deformation, and reveals that collapse initiates once a critical surface curvature is exceeded.

To address the inverse problem of reconstructing a nanoframe’s cross-sectional profile from atomistic data, we developed a geometric reconstruction algorithm that projects the three-dimensional atomic configuration onto a two-dimensional meridional plane. This approach parametrizes the nanoframe as a figure of revolution and uses spline-based contour reconstruction to obtain a smooth and noise-robust description of the cavity and outer surface. The method draws upon mathematical tools used in electron tomography,^43,44^ mesh reconstruction,^45^ and medical imaging,^46^ and is computationally efficient for large-scale atomistic data. Software implementations of similar numerical steps—edge detection, binning, and spline fitting—are described in ref 50.

### 2.2 Shape Parametrization and Profile Reconstruction Algorithms

The algorithm for classifying the shape of a nanoframe with a rotational image around the *Y*-axis consists of the following steps:

1. Preparing the point cloud. From the simulated data, we obtain an array of atomic coordinates or grid nodes {(*x*_*i*_, *y*_*i*_, *z*_i_), *i* = 1··· N}. We assume that the system’s center of mass is already shifted to the origin (0, 0, 0). We select small rotation angles so that the rotation axis coincides with the *Y*-axis.
2. Projection onto a profile plane. Each three-dimensional point (*x_i_*, *y_i_*, *z*_*i*_) is projected onto the Y−ρ plane, where the *Y*-axis is the vertical axis and the ρ-axis is the radial distance to this axis. The coordinates of a point in this plane are calculated as follows: the Y coordinate is equal to *y_i_*; the ρ-coordinate is equal to ρ_i_ = (*x_i_* ^2^ + *z_i_* ^2^)^1/2^.

The resulting set of points (*y*_i_, ρ_*i*_) represents a meridional cross-section of the nanoparticle, from which its profile can be reconstructed.

3. Binning by the *y*-coordinate. The *y*-range is divided into M intervals (bins). In each bin *k*, we collect all points for which *y_i_* falls within this interval and find two values: upper profile *R_k_* = max {ρ_i_ | *y_i_* in bin *k*}; lower profile *L_k_* = min {ρ_i_ | *y_i_* in bin *k*}. If a bin contains no points, then it is omitted in the subsequent approximation. The *y_k_* coordinates become the centers of the bins.

4. Spline approximation of profiles. Two smooth one-dimensional splines, *H*(*y*) and *L*(*y*), are constructed from the sets of nodes {*y_k_*, *R_k_*} and {*y_k_*, *L_k_*} (from Figure 1 meaning *H*(*y*) and *L*(*y*) are clearly understood). The smoothing parameter *s* of the spline is adjusted depending on the noise and the desired smoothness: when *s* = 0, the spline passes through all the nodes (interpolation), while when *s* > 0, it produces a smoother approximation. The maximum bound for *s* is determined by the formula:

$$s_{\max}=M+{\left(2\cdot M\right)}^{1/2}$$

**Figure 1:**
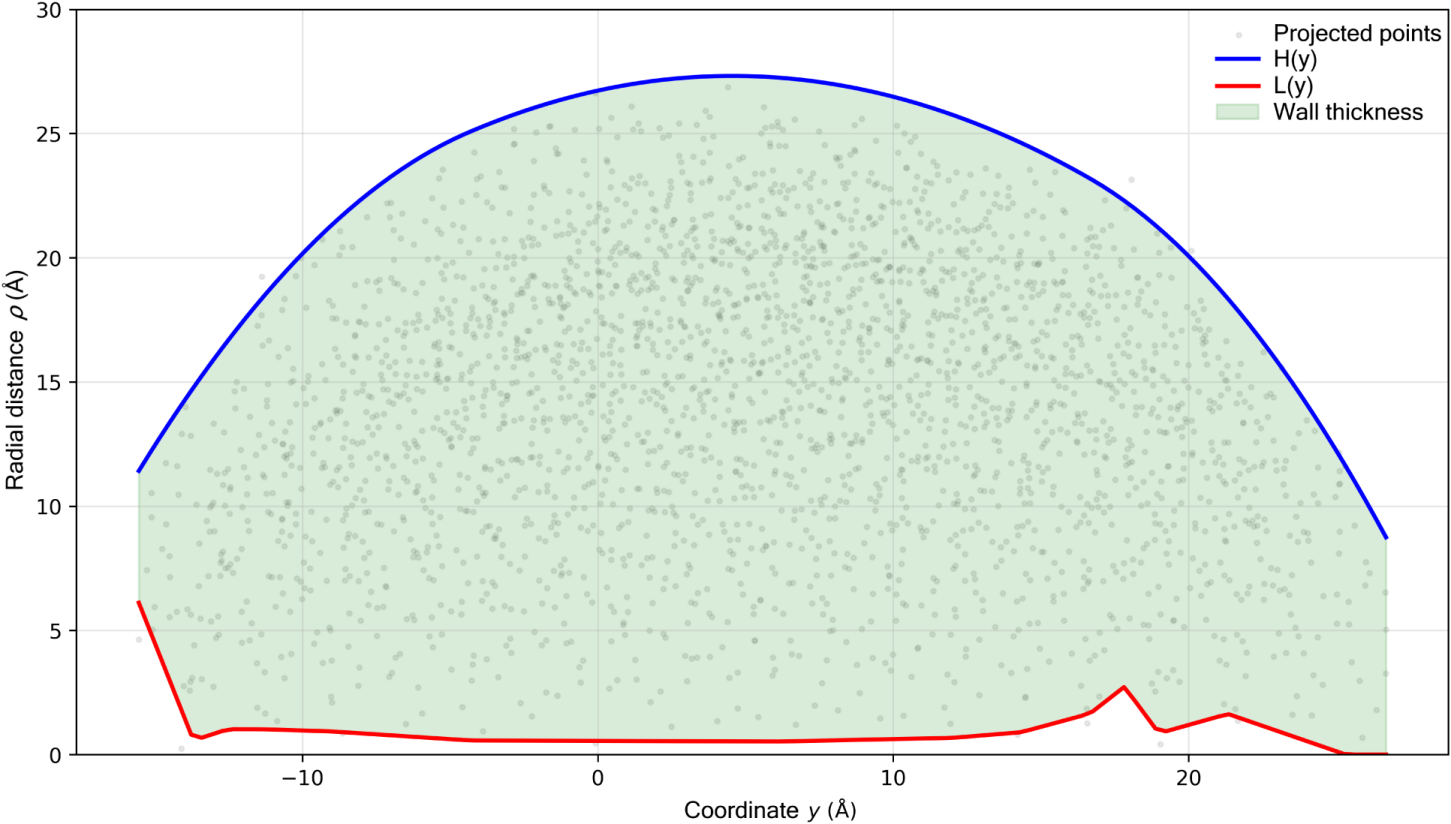
To the methodology of constructing profiles *H*(*y*) and *L*(*y*).

The spline smoothing parameter *s* was selected based on point-cloud density and thermal noise level. For low-temperature configurations (*T* < 600 K), *s* = 0 was used to preserve full geometric fidelity, while at higher temperatures (*T* ≥ 600 K) values in the range 0.1·*s*_max ≤ *s* ≤ 0.3·*s*_max were applied to suppress thermally driven outliers without altering cavity topology. This choice stabilizes the numerical derivatives *H*′(*y*), *L*′(*y*) while retaining correct pore geometry. We verified that variations of *s* within this range do not affect state classification (classic/concave/closed/collapsed).

5. Numerical integration of profile properties. On a dense uniform grid *y* ∈ [*y*_min_, *y*_max_], we calculate the following parameters: the values of *H*(*y*), *L*(*y*), and their derivatives *H*′(*y*), *L*′(*y*); the 2D cross-sectional area of the nanoframework

S=∫{ymin}^{ymax}[H(y)−L(y)]dy

The length of the closed contour (the “perimeter” of the cross-section)

$$P=\int {\left(1+{[H^{\prime }\left(y\right)]}^{2}\right)}^{1/2}dy+\int {\left(1+{[L^{\prime }\left(y\right)]}^{2}\right)}^{1/2}dy$$

Here, the flattening parameter *f* quantifies the deviation of the cross-sectional profile from rotational symmetry and can be interpreted as a measure of stress-induced anisotropic deformation. In the context of collapse dynamics, increasing *f* reflects curvature redistribution and wall thinning prior to structural failure. We observe that *f* approaches a threshold (0.18–0.22) immediately before cavity collapse across all studied geometries, indicating its role as a universal instability precursor. The integrals are calculated using Simpson’s method or another suitable numerical quadrature.

6. Classification of shape by pore state. The *H*(*y*) profile of the external surface is always convex upward. Using the *L*(*y*) profile, four possible nanoframe states are distinguished: A) "Collapsed", if max (*L*(*y*)) *L*_thresh_, i.e., the entire lower contour lies within the threshold radius *L*_thresh_ → the entire structure is effectively flattened. B) "Closed", otherwise, if both ends of the profile *L*(*y*_min_) and *L*(*y*_max_) are less than *L*_thresh, both pores are closed, but in the central part *L*(*y*) is still above *L*_thresh_. C) "Classic" (classical torus), if no pores are closed (*L*(*y*_min_) and *L*(*y*_max_) ≥ *L*_thresh_), and both ends of the profile lie at the same height or above the central cross-section:
$$L\left(y_{\min}\right)\geq L\left(y_{\mathrm{mid}}\right)\;\mathrm{and}\;L\left(y_{\max}\right)\geq L\left(y_{\mathrm{mid}}\right)$$

D) “Concave” (concave torus), if all other cases where the pores are not collapsed, but at least one end *L­(y*_min_) or *L*(*y*_max_) is below the central level *L*(y_mid_).

Here, *y*_mid_ = (*y*_min_ + *y*_max_)/2. For the above comparisons, the threshold value *L*_thresh_ is chosen based on the physical problem (e.g., *L*_thresh_ ≈ the interatomic distance or 1 Å).

The results of the proposed algorithm are: (i) two smooth profiles, *H*(*y*) and *L*(*y*), defining the precise shape of the cross-section; (ii) numerical values of the area *S* and perimeter *P*; and (iii) a label for the porous structure type {collapsed, closed, classic, concave}.

Advantages of the method compared to surface triangulation:

Clear parametrization of critical geometric parameters. Wall thickness *h*, cavity radius *R*_c_, and the radius (and curvature) of each pore are explicitly specified using spline parameters.
Automatic detection of nanoparticle rotation and identification of the symmetry axis.
Robustness to noise and surface defects. Artifacts do not mask the true collapse/closure of the pore.
Direct analysis of the four collapse stages using numerical parameters.
The profile of the 2D shape is sufficient to estimate the size of the cavity, each pore, as well as the total surface area and volume of the nanoparticle.

## 3 Results of Atomistic Simulations

### 3.1 Caloric Curves and Transition Temperatures

Following^2,42^ the analysis, the structural transformations and thermal stability of nanoframes were examined using the temperature evolution of the potential component of the specific internal energy. Figure 2 presents representative caloric dependences for nanoframes of different cavity sizes; the corresponding initial configuration is shown in Figure 3 (the nanoframes have a central cavity and two porous walls opposite each other, containing 2700 atoms). The characteristic wave-like features observed in the potential energy within the pore-degradation interval are consistent with earlier atomistic studies of porous nanosystems.^6^

**Figure 2:**
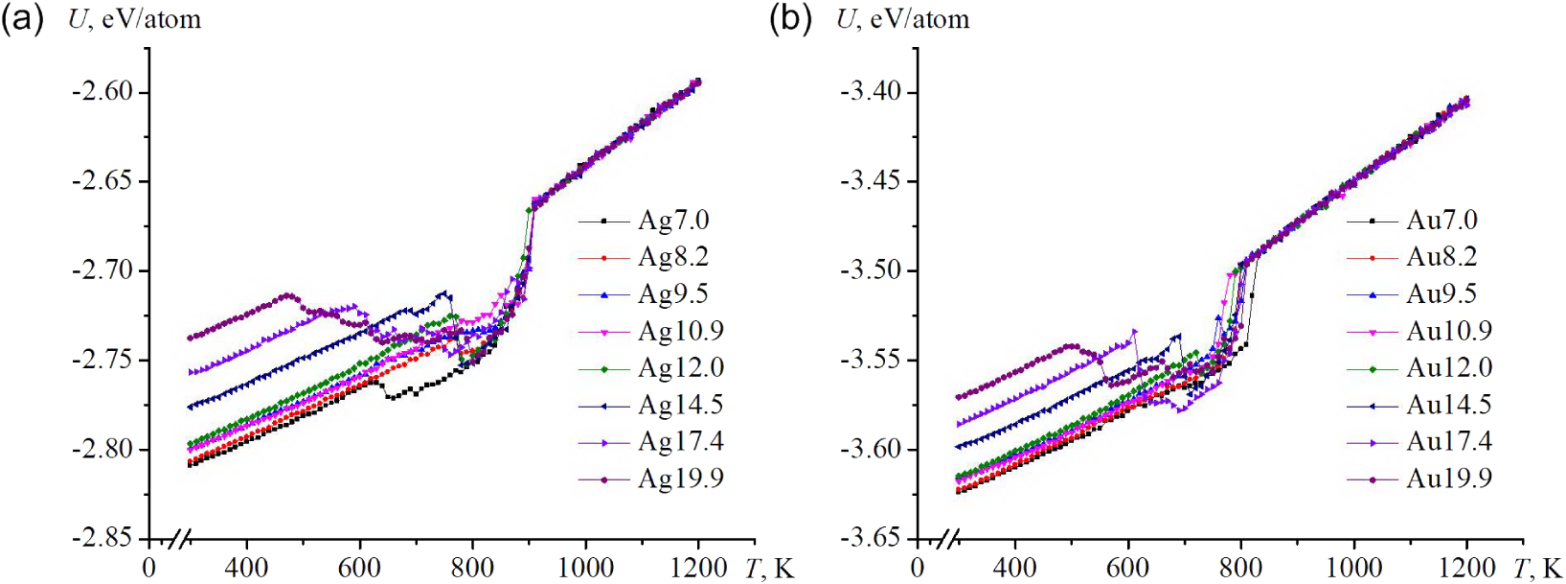
Typical temperature dependences of the potential part of the internal energy for nanoframes of different sizes: (a) Ag, (b) Au. The legend indicates the internal sizes of the cavity of the nanoframes (in Å).

**Figure 3:**
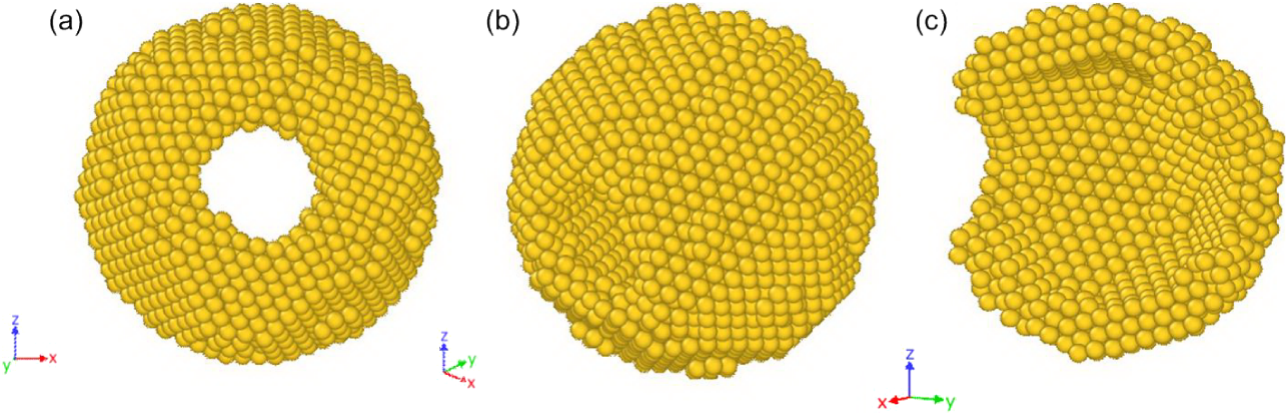
The typical initial configuration for the simulation of the nanoframe with the internal size *R*_in_ = 19.9 Å at a temperature of 300 K is shown in different projections (a, b) and in section (c) (visualization in Ovito^42^).

### 3.2 Crystallinity Evolution

The caloric curves exhibit several distinct inflection points that correspond to structural rearrangements, including sequential healing of lateral pores and the formation or annihilation of vacancies, persisting up to the melting temperature. Melting is identified by a sharp discontinuity in the caloric curve, after which the potential energy increases nearly linearly with temperature. At lower temperatures, linear portions of the caloric dependence define the thermal stability ranges of the nanoframes. After melting, the specific internal energies of Ag nanoframes become nearly identical, whereas for Au, the difference does not exceed ∼0.01 eV per atom. Table 2 summarizes the characteristic temperatures associated with external-pore closure, collapse of the internal cavity, onset of the first structural transition, maximum crystallinity, and melting.

**Table 2:** Temperatures of Structural Transformations and Phase Transitions for Nanoframes of Different Sizes

	Temperature, K					
R_in_, Å	1 Hole closed	2 Holes closed	Hollow closed	First local transition	Maximum crystallinity	Melting
Ag						
7.0	645	655	665	590	750	900
8.2	760	765	790	605	730	900
9.5	743	785	803	690	750	890
10.9	780	790	825	690	755	880
12.0	760	780	793	655	790	900
14.5	755	764	783	720	800	892
17.4	605	623	844	490	770	852
19.9	605	642	800	445	805	875

	Temperature, K					
R_in_, Å	1 Hole closed	2 Holes closed	Hollow closed	First local transition	Maximum crystallinity	Melting
Au						
7.0	662	685	823	750	480	825
8.2	680	686	810	770	603	815
9.5	705	720	740	720	540	785
10.9	705	715	770	725	470	772
12.0	690	710	715	705	585	805
14.5	665	678	688	670	710	802
17.4	618	620	720	615	685	795
19.9	585	590	730	520	705	805

Terminology explanation: (i) “1 hole closed” denotes a lateral pore vanishing event; (ii) “2 holes closed” denotes simultaneous closure of both lateral pores; (iii) “hollow closed” denotes collapse of the internal cavity. All three transitions are detected from geometric descriptors (Section 2.2) and do not imply melting. (iv) “first local transition” is the temperature at which the fluctuation in the proportion of any crystalline phase first exceeds 5% relative to the value at 300 K; (v) “maximum crystallinity” is the temperature at which the maximum total proportion of fcc+hcp is reached. For parameters (iv) and (v), the average value over the series of experiments is used.

The thermal stability maps for all systems are provided in Figure 4. These maps allow predictive determination of the key transition temperatures: closure of external cavities, collapse of the inner hollow, the temperature of the first structural transition, the temperature of maximum crystallinity, and the melting point. For gold nanoframes with small cavity sizes of less than 1.45 nm, no recrystallization is observed. The graph of the proportion of the crystalline phases is quite flat ­(Figure 6). The maximum crystallization temperature is determined approximately; in all the cases mentioned, this value is unrelated to (and lies below) the pore closure temperature. Silver nanoframes behave differently, and pore closure is accompanied by recrystallization. The trends observed in Figure 4 are directly relevant for predicting the optical response of Au nanoframes,^51^ since the ratio of lateral size to wall thickness affects the scattering spectra of individual nanoframes (see Figure 5). This experimental study is closest to our research objects in terms of object configurations. Furthermore, Figure 5 shows the size range of the objects and the change in the scattering spectrum. Thus, it becomes clear why, in our case, we chose nanoframes with different internal radii but with a constant number of atoms. Additionally, the degradation sequence revealed in Figure 4 shows conceptual similarity to the photothermal transformation cycle modeled in ref^52^.

**Figure 4:**
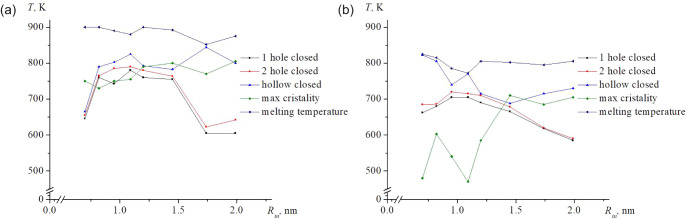
Temperature stability map of nanoframes of different sizes is as follows: (a) Ag, (b) Au.

**Figure 5:**
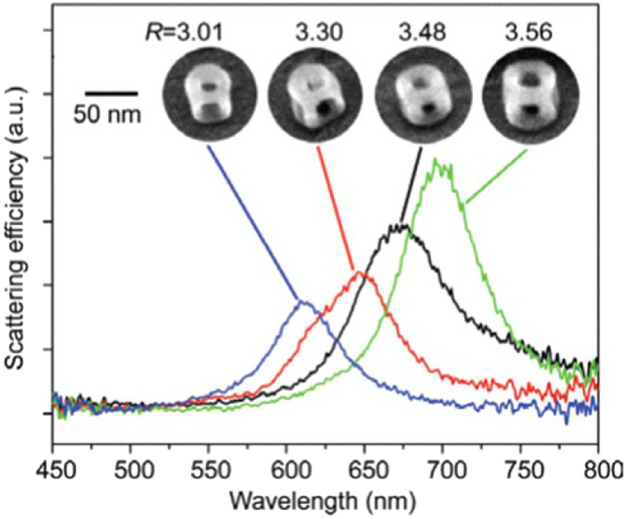
Scattering spectra of individual Au nanoframes and SEM images of the corresponding nanoframes are provided. Here, *R* = *l*/*t*, where *l* is the outer edge length of the nanoframe, *t* is the thickness of the nanoframe walls (Reproduced from ref 51. Copyright [2008] American Chemical Society).

To complement this thermodynamic analysis, the evolution of local atomic order was examined using OVITO.^42^ Figures 6 and 7 display the temperature dependences of atoms with *fcc*, *hcp*, *bcc*, and icosahedral local symmetries. For all nanoframe sizes and both metals, the fcc and hcp motifs dominate throughout the stability interval. Typically, their temperature profiles exhibit three regions: a low-temperature plateau, a broad maximum (sometimes weakly expressed), and a subsequent abrupt decline. In some cases, this interval spans up to ∼300 K. The envelopes of the *fcc*-*hcp* fractions display similar shapes for all systems. Additional local structures are also present: bcc-like coordination appears after the hollow-closure temperature (indicated by dotted lines in Figures 6 and 7), consistent with prior observations of high-temperature *bcc* formation in nanoscale *fcc* systems, while icosahedral nuclei emerge predominantly near the melting region. Notably, the temperature of cavity closure often aligns with the maximum crystallinity, although several nanoframes (Figure 7a, b, and d) exhibit the closure point near a rapid decrease in crystalline order, reflecting geometry- and composition-dependent relaxation pathways. Ordered local crystalline phases are identified before the nanoparticle transitions to a liquid state. Thus, the diagrams include the temperature range from the onset of heating to the end of melting.

**Figure 6:**
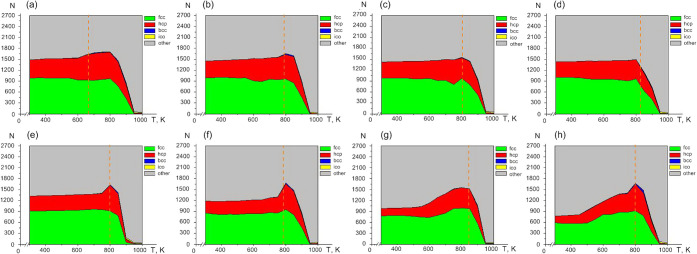
Temperature dependences of the number of atoms with an identified type of local structure (*fcc*, *hcp*, *bcc*, *ico*, or unrecognized structure) of different sizes of silver nanoframes are shown, with the initial size of the internal cavity indicated as follows : (a) 7.0 Å, (b) 8.2 Å, (c) 9.5 Å, (d) 10.9 Å, (e) 12.0 Å, (f) 14.5 Å, (g) 17.4 Å, (h) 19.9 Å. The hollow-closed temperature is indicated by a dotted line.

**Figure 7:**
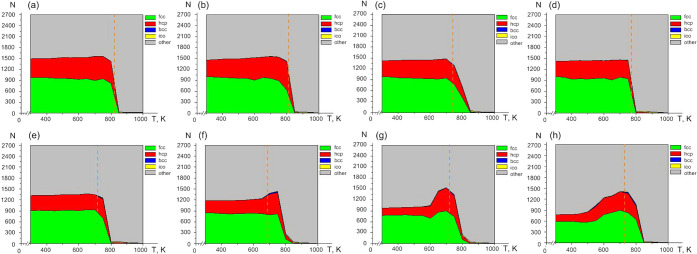
Temperature dependence of the number of atoms with an identified type of local structure (*fcc*, *hcp*, *bcc*, *ico*, or unrecognized structure) of different sizes of gold nanoframes (the initial size of the internal cavity is indicated): (a) 7.0 Å, (b) 8.2 Å, (c) 9.5 Å, (d) 10.9 Å, (e) 12.0 Å, (f) 14.5 Å, (g) 17.4 Å, (h) 19.9 Å. The hollow closed temperature is indicated by the dotted line.

### 3.3 Cavity Collapse Kinetics

The temperature dependence of the internal cavity radius, shown in Figure 8, provides further insight into collapse dynamics. Each curve displays a characteristic Z-shape: (i) a low-temperature interval of thermal stability; (ii) a sharp decline corresponding to cavity collapse; and (iii) a high-temperature region associated with the molten nanoparticle. As cavity size increases, the stability interval narrows, reaching approximately 150 K for the largest Ag nanoframe and ∼200 K for the corresponding Au structure.

**Figure 8:**
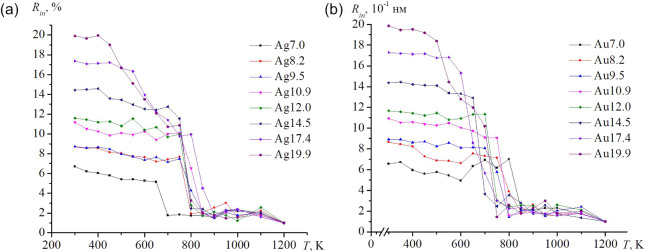
Temperature dependences of the internal radius *R*_in_ of various nanoframes are presented, with the initial size of the internal cavity indicated as follows : (a) Ag, (b) Au.

The flattening parameter, an important descriptor of the pore-surface shape, is shown in Figure 9. The data for all sizes exhibit a clear linear trend with temperature and well-defined upper and lower boundaries, particularly below ∼600 K for Ag and ∼700 K for Au. However, some outliers do exist. In this case, the overall point cloud is shown, and there is no differentiation by pore size. Silver nanoframes at temperatures of 700 K and above exhibit a greater spread in morphologies. However, the asphericity (a positive measure of *f*) was present for all nanoframes studied. A similar temperature-dependent distribution of the external radius perpendicular to the symmetry axis is presented in Figure 10. Despite an overall linear growth with temperature, a slight decrease near the melting region is observed, reflecting a reduction in the total volume associated with pore collapse. This effect is most clearly visible in Figure 9b.

**Figure 9:**
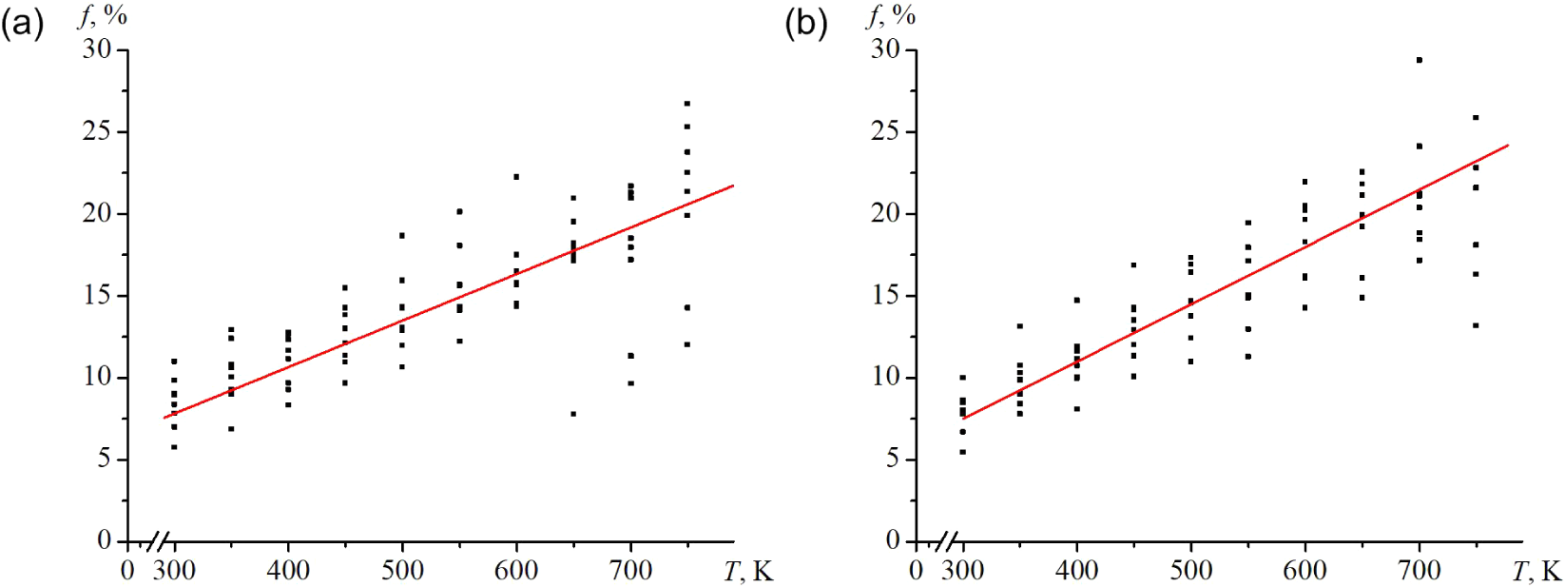
Temperature dependences of the flattening parameter of nanoframes are shown, with the cloud of values for all sizes in the given temperature range provided: (a) Ag, b – Au. The red line determines the trend of change in the flattening parameter.

**Figure 10:**
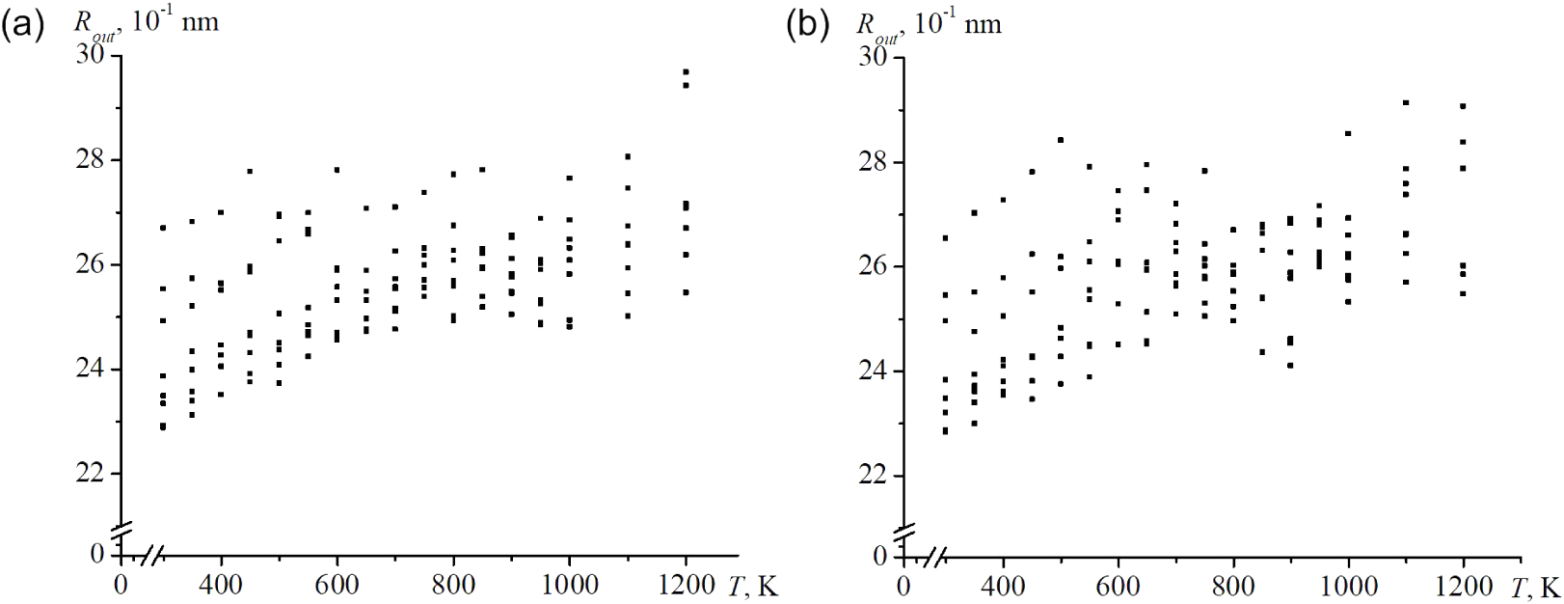
Temperature dependences of the external radius of nanoframes perpendicular to the axis of symmetry are shown, with a cloud of values for all sizes provided: (a) Ag and b) Au.

### 3.4 Pore Evolution and Surface Concavity

An analogous three-stage pattern appears in the temperature dependence of the pore volume fraction (Figure 11). The first interval occupies a narrow temperature range in which the pore volume remains nearly constant; the second interval corresponds to dynamic pore healing; and the third interval marks the melting transition. This provides an additional method for estimating melting temperatures, complementing caloric-curve analysis,^2,49,53–55^ heat capacity profiles,^53,55,56^ and structural descriptors.^55^

**Figure 11:**
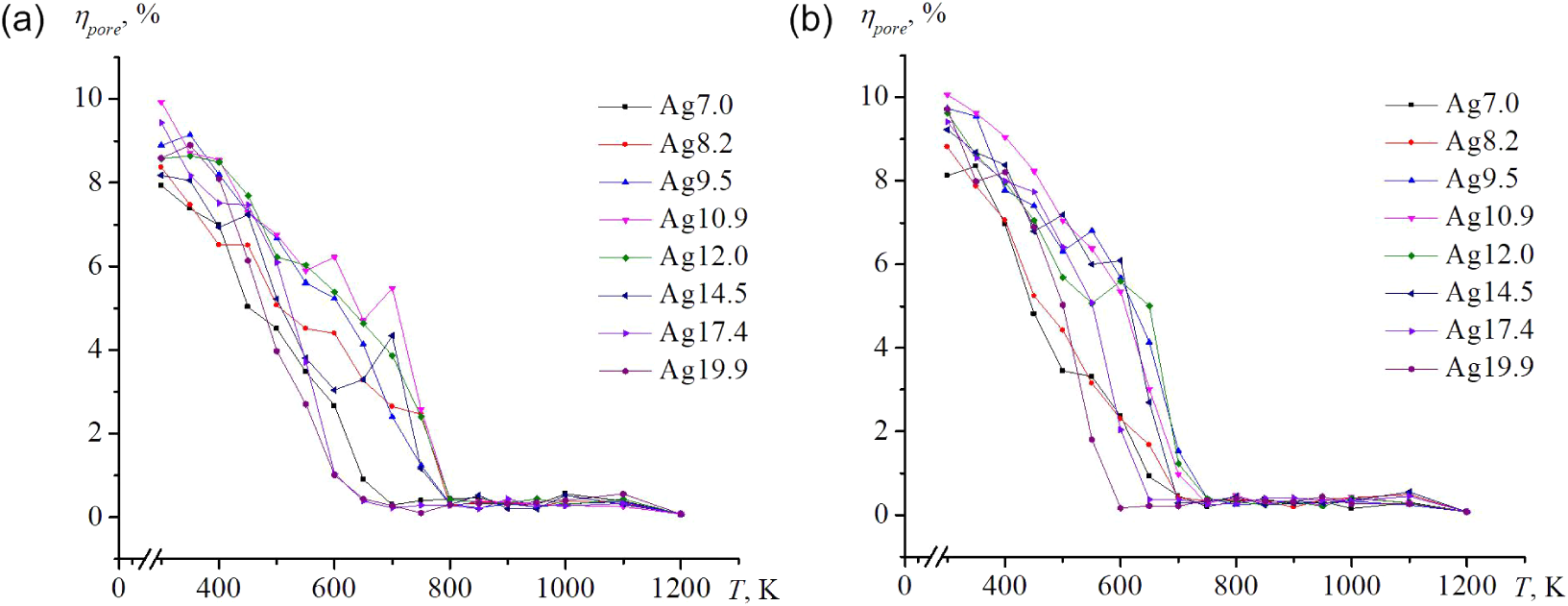
Temperature dependences of the proportion of pores in nanoframes are presented, with the initial size of the internal cavity indicated as follows : (a) Ag and (b) Au.

A more intricate relationship is observed in the dependence of the concavity metric *C*(*S*_pore_) on the pore area (Figure 12). When analyzed from right to left along the abscissa, the curves first increase—linearly for large cavities and more steeply for small ones—followed by a local maximum. This maximum corresponds to the attainment of a critical concavity threshold that initiates cavity collapse. Variations among cavity sizes reflect geometry-specific atomic rearrangements occurring during structural degradation.

**Figure 12:**
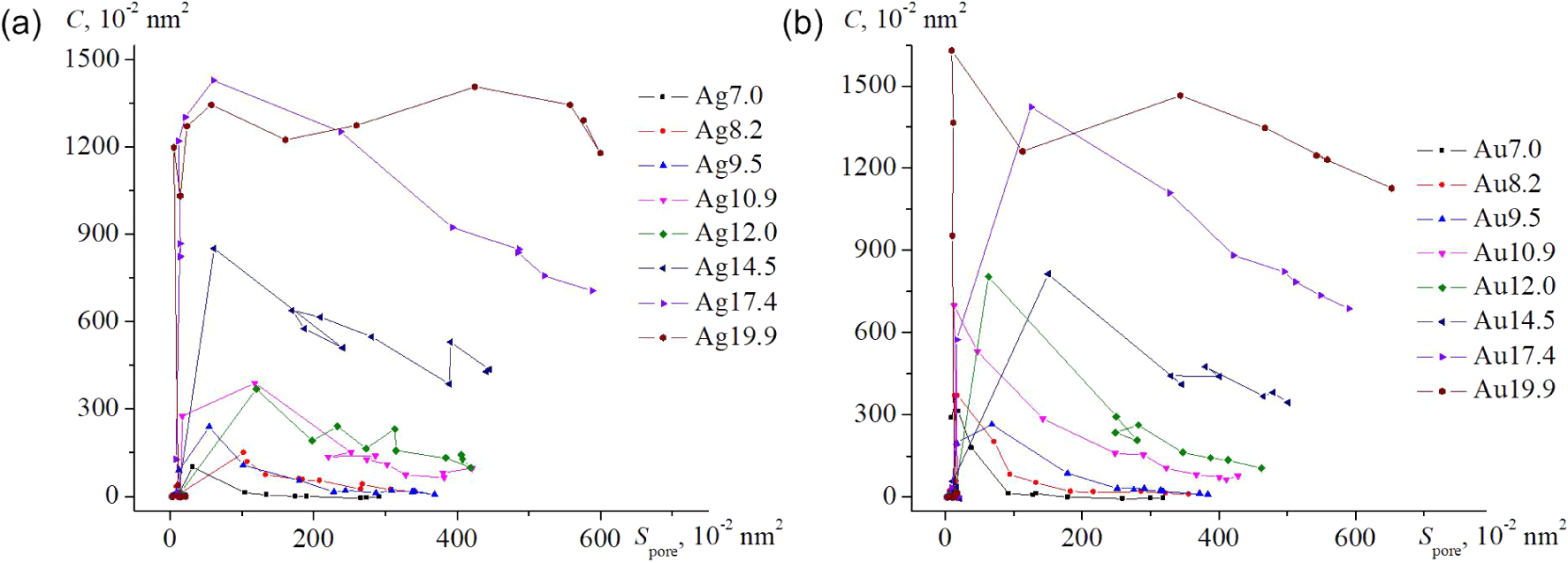
Dependence of the concavity of the inner surface of nanoframes on the pore area is shown, with the initial size of the internal cavity indicated as follows : (a) Ag and (b) Au.

Pore collapse is not the “final collapse” but rather a key stage of relaxation and hardening of the nanostructure. In terms of functional properties (catalytic activity and surface area), pore collapse is certainly degradation. However, from the point of view of atomic thermodynamics and stability of the nanosystem, it is a positive relaxation process.

Figure 13 shows in more detail the process of restructuring the internal structure and the dual surface of a hollow nanoparticle: when the pores are closed, as well as just before the moment of complete collapse of the internal cavity. It should be remembered that we are dealing with an ultrathin frame “cut out” of a large icosahedron. We conducted preliminary simulations to compare the thermodynamic stability of hollow nanoparticles “cut out” from an icosahedron and a single crystal. The icosahedron is a deliberate and justified choice for this size range, which in itself is already a defective, stressed structure with multiple twin boundaries between *fcc* grains. In this context, the mechanism of the time-combined relaxation + recrystallization process can be visually described as “resource redistribution”.

**Figure 13:**
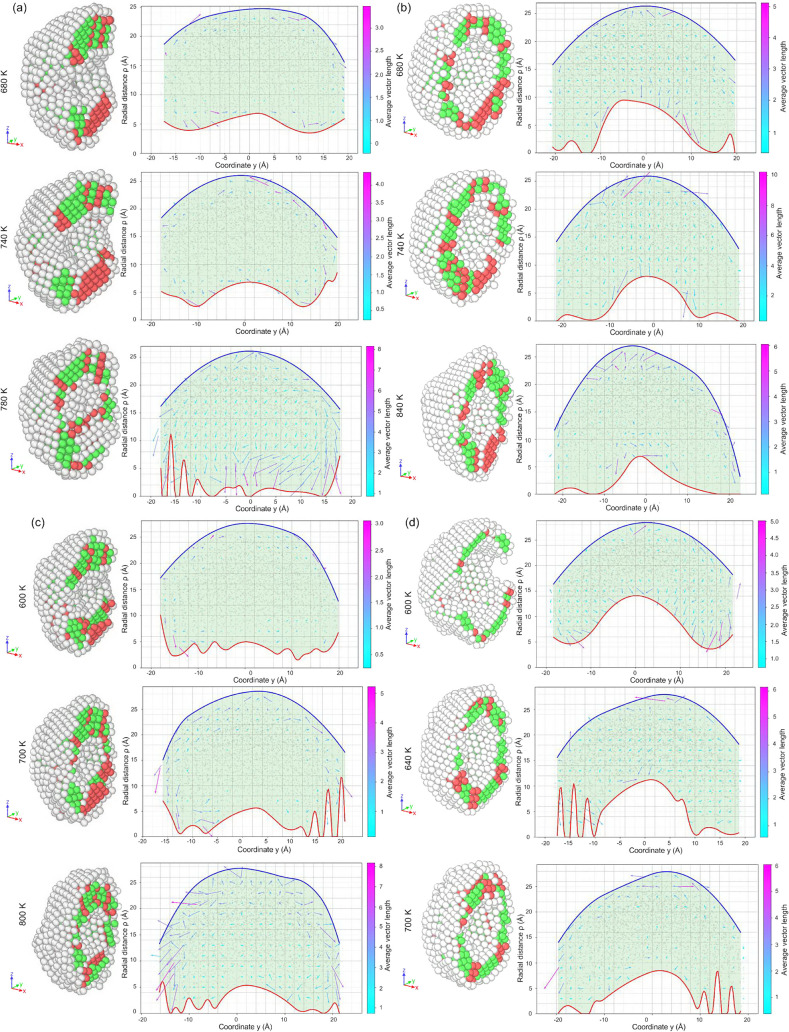
(a) Visualization of the hollow-closed process for Ag nanoframes with *R*_in_ = 8.2 Å. On the left are 3D configurations (in cross-section) with the PTM identifier applied to recognize crystalline phases (color coding corresponds to Figures 6 and 7) and on the right are 2D projections with collective relaxation vectors. The red line represents the inner surface of the cavity; the blue line represents the external nanoparticle surface. (b) The same visualization is provided for Ag nanoframes with *R*_in_ = 17.4 Å. (c) The same visualization is provided for Au nanoframes with *R*_in_ = 8.2 Å. (d) The same visualization is provided for Au nanoframes with *R*_in_ = 17.4 Å.

The collapse of cavities and lateral pores in ultrathin nanoframes is a thermodynamically favorable relaxation pathway, driven primarily by the reduction of surface energy and redistribution of curvature. Regions with high local curvature, particularly concave inner surfaces near pore mouths, possess elevated surface energy due to the strong deviation of atomic coordination from the equilibrium *fcc* environment. Their gradual elimination during heating constitutes a direct relaxation toward a lower-energy configuration. Each pore-closure event removes an area of extreme curvature and simultaneously reduces internal stress concentrations, thereby stabilizing the surrounding lattice.

### 3.5 External Deformation and Flattening Metrics

Pores also function as geometric defects within the ultrathin, icosahedrally derived framework. Their presence enhances stress localization and perturbs the local bonding environment. During thermal activation, partial recrystallization and localized plastic deformation increase the packing density in these regions, strengthening the wall and promoting further smoothing of the internal surface. This behavior is consistent with the thermodynamic tendency of nanoparticles to transition from metastable, high-curvature geometries to more compact, energetically favorable forms.

The relaxation pathway is strongly influenced by structural inheritance from the parent icosahedral architecture, which contains intrinsic strain, twin boundaries, and coordination defects. Upon heating, these defects become mobile and redistribute across the framework. Rather than disappearing, they accumulate at high-energy sites, such as concave pore edges and convex ridges. These defect-enriched zones become nuclei for subsequent deformation. Such behavior is consistent with the known role of defect-mediated rearrangement in *fcc*-derived nanostructures and with the distribution of tetrahedral and octahedral interstices in dual *fcc* lattices.^57^

Because the nanoframe consists of narrow, interconnected struts instead of a continuous shell, geometric evolution requires redistribution of mass across the entire framework. This leads to inherently nonuniform collapse dynamics that proceed through a sequence of localized events instead of a global, simultaneous transformation. The process resembles iterative smoothing: the system reduces the most energetically unfavorable curvature first and then propagates the remaining stress and defect flux toward the next unstable region.

This behavior manifests in the asymmetric and cyclic pore evolution observed in Figure 13. The simulations reveal a consistent pattern: narrowing of one lateral pore (*L*) induces curvature reduction and defect accumulation that lowers local energy, but also generates a stress redistribution across the framework. Because the frame is mechanically anisotropic and extremely thin, these stresses cannot be absorbed uniformly. Instead, they propagate toward the second pore (*R*) or another high-curvature site. This results in temporary deformation there, followed by partial relaxation and subsequent return of the deformation front to the original site. Such alternating localization of deformation is evident in Figure 13a for Ag nanoframes and in Figure 12c,d for Au nanoframes.

In the case of icosahedrally derived frames, the relaxation thus corresponds to a sequential elimination of energetically unfavorable geometric motifs, sharp pore edges, concave regions, and inherited defect clusters, and the gradual rearrangement of the ultrathin atomic network toward a less curved, more stable configuration. The extreme thinness and geometric complexity of the frame prevent simultaneous melting–recrystallization of the entire volume; instead, the system relaxes through a series of localized curvature-reduction steps, each accompanied by defect migration and partial recrystallization. This mechanistic picture fully explains the observed asymmetric profile deformations and the alternating cooperative atomic motions documented in Figure 13.

Experimental data confirm the “mobility” and asymmetry of the nanoframe walls, which can be caused by both the technology used to produce the nanoframes (for example, using the galvanic replacement technique)^58^ and thermodynamic factors. An example of SEM images is shown in Figure 14.^58^

**Figure 14:**
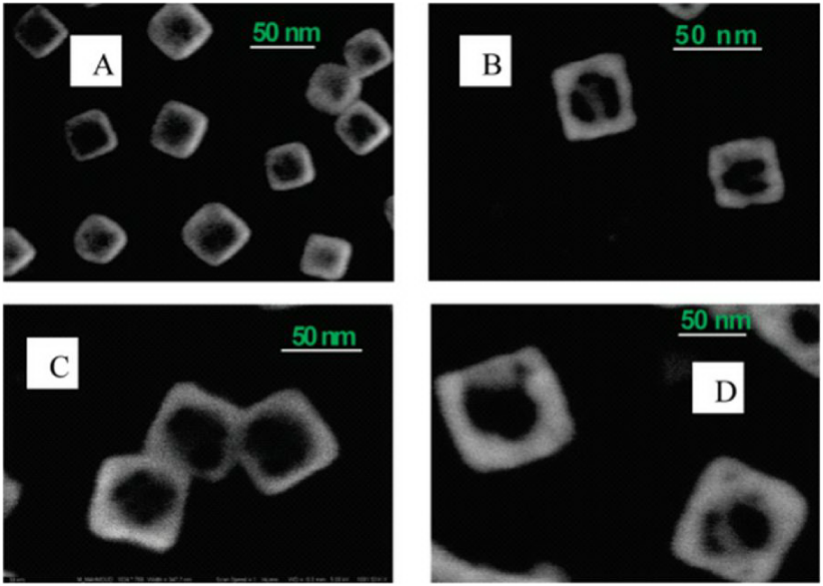
SEM images of Au nanoframes with wall lengths of (A) 35, (B) 42, (C) 51, and (D) 83 nm, and wall thicknesses of 10, 9, 10, and 19 nm, respectively (reproduced from ref 58, Copyright [2010] American Chemical Society).

The transition ranges reported here are directly relevant for plasmonic and catalytic applications, where structural porosity governs both hot-spot formation and active surface area.^59,60^ For example, the external pore-closure temperature defines the upper photothermal operating limit, while cavity collapse marks the loss of an accessible internal surface, which is detrimental for SERS and catalytic enhancement. Thus, the derived maps can be used as predictive stability guidelines for device-level thermal cycling.

## 4 Conclusion

This study established a quantitative framework for understanding the thermal stability of hollow noble-metal nanoframes and provided robust, general criteria for predicting their structural evolution. By integrating the tight-binding potential in Monte Carlo simulations with a universal shape-parametrization method capable of reconstructing cavity and pore geometries from atomic coordinates with ångström-level precision, we obtained a material-independent description of pore closure, cavity collapse, and global deformation. The methodology is applicable to nanoframes of different sizes, porosities, symmetries, and compositions, underscoring its broad utility for analyzing complex hollow nanostructures.

The simulations reveal distinct but quantifiable relaxation pathways for the Au and Ag systems. Silver nanoframes undergo lateral pore closure at 605–785 K and cavity collapse at 783–844 K, while gold nanoframes close pores at 585–720 K but resist collapse until 688–823 K, reflecting their more coordinated recrystallization dynamics. Across all architectures, the internal radius decreases by 6–10 Å within a narrow 30–50 K interval, marking a sharp collapse threshold. The flattening parameter consistently rises to 0.18–0.22 immediately before global deformation, demonstrating its value as a universal geometric predictor of instability.

The present simulations neglect (i) mechanical loading, (ii) chemical environment, and (iii) multicomponent diffusion and assume pairwise tight-binding interactions. These simplifications enable isolation of thermally driven relaxation but do not capture corrosion, oxidation, or ligand-mediated stabilization. Future extensions may incorporate reactive potentials, solvent interactions, or external stresses to expand the applicability to experimentally relevant conditions.

Beyond revealing material-specific mechanisms, the quantitative stability maps and geometric descriptors developed here constitute a predictive and transferable toolkit for the engineering of thermally robust hollow nanostructures. These results provide actionable design rules—such as identifying safe thermal operating windows, selecting wall thickness and porosity to suppress premature collapse, and diagnosing instability through shape metrics. Such predictive capability is directly relevant for applications in heterogeneous catalysis, plasmonic and photothermal energy conversion, chemical and biological sensing, and any technologies where hollow metal-based nanoframes experience elevated temperatures or repeated thermal cycling.

## Data Availability

The data and final model used in this study are available at the file-sharing service: https://disk.yandex.ru/d/gF4Ds4x7EMh3MA.
